# Editorial: The role of the gut microbiota on bone mass in health and disease

**DOI:** 10.3389/fendo.2023.1346156

**Published:** 2024-01-05

**Authors:** Sadiq Umar, Owen Cronin, Abdul Malik Tyagi

**Affiliations:** ^1^ Department of Oral Biology, College of Dentistry, University of Illinois, Chicago, IL, United States; ^2^ School of Medicine, University College Cork, College, Road, Ireland; ^3^ Department of Rheumatology, Bon Secours Hospital, Cork, Ireland; ^4^ Division of Orthopedic Surgery, Washington University, Saint Louis, MO, United States

**Keywords:** gut microbiota, bone mass, osteoporosis, osteomicrobiology, intestine

The microorganisms colonizing the digestive tract consist of almost 100 billion fungi, bacteria, and bacteriophages that play a vital function in various aspects of human health, including bone health. While the connection between gut microbiota and bone health is an area of ongoing research, there is strong evidence to suggest that the composition and activities of gut bacteria can influence bone density and overall bone health in several ways, both in healthy and diseased individuals. Data from animal studies suggest that harboring beneficial bacteria helps achieve peak bone mass at a young age, allowing individuals to sustain healthy bone longer in old age. Several Studies from mouse models of postmenopausal osteoporosis showed that colonizing lactobacillus bacteria protected sex steroid-deficiency-induced bone loss (1–5).

This Research Topic includes some pertinent reviews and original research articles on the ongoing efforts to inspect the influence of gut microbiota on bone mass regulation. This editorial highlights the discoveries of all the articles included in this Research Topic. Zhou et al. examined the effect of the natural compound Eleutheroside E (EE), obtained from the palatable therapeutic herb *Acanthopanax senticosus*, on sex steroid deficiency-induced bone loss. They identified ten targets from the docking experiment and confirmed these results from an *in-vivo* experiment. They showed decreased serum levels of pro-inflammatory osteoclastogenic cytokines such as TNF-α, TRAP, CTX, IL-6, and LPS. The author also reported that the serum levels of P1NP were higher in EE-treated mice. Zhou et al. further showed that Lactobacillus bacteria increased with EE treatment. However, EE treatment decreased the frequency of Clostridiaceae.

Okoro et al. explored the association between the human intestinal microflora and scans of the radius and tibia by high-resolution peripheral quantitative computed tomography (HR-pQCT) in two big groups of patients. The author accessed data from 1227 subjects ranging from 32 – 89 years from Framingham Heart Study (FHS), and data from the second study group was accessed from 836 subjects ranging from 78-98 years from osteoporosis in Men Study (MrOS). Okoro et al. reported that 37 bacterial genera in FHS and four bacterial genera in the MrOS study were associated with bone mass, such as the association of DTU089 bacterial strain with bone mass. Results from meta-analysis revealed that a higher abundance of Akkermansia was associated with a lower total radius vBMD. Similarly, the higher abundance of DTU089 bacteria was associated with lower tibia cortical vBMD.

Furthermore, the higher frequency of the Lachnospiraceae NK4A136 bacteria and Faecalibacterium bacteria were associated with higher tibia cortical vBMD. The author further investigated the functional association of metabolic pathways with the changes in microbial abundance and bone phenotypes in each cohort. They found eight pathways, including the super-pathway of histidine, purine, and pyrimidine biosynthesis, associated with bone measures of the tibia cortical compartment. In conclusion, these findings suggest that there is likely a link between the gut microbiome and skeletal metabolism.

Most studies conducted in humans are association studies; here, Li conducted a study between gut microbiota and bone mineral density (BMD). The data showed that specific genera of bacteria, including Prevotella 9, were positively associated with higher BMD. Li’s data suggested a causal relationship between gut microbiota and BMD and identified specific bacteria taxa that regulate bone mass variation. Jackova et al. investigated and analyzed the particular shifts in gut microbiota related to ovarian sex hormone changes induced by oophorectomy and subsequent hormonal therapy in women. The data showed no significant changes in gut microbiota composition six months after oophorectomy despite substantial changes in hormone levels, BMD, and bone metabolism. Hormonal therapy after oophorectomy prevented bone loss but only marginally affected gut microbiota. There were no significant differences in β-diversity related to hormonal status. Body mass index (BMI) was the most significantly associated with microbiota variance. Results from this study showed that microbiota was not a suitable predictive factor for bone metabolism.

Sun et al. summarize the effect of traditional Chinese medicine on the regulation of 5-hydroxytryptamine (5-HT). 5-HT is a neurotransmitter closely associated with bone tissue metabolism. Sun et al. highlighted that Traditional Chinese Medicine can regulate 5-HT, thereby regulating bone mass. Bailey and Fraser, in their report, explored the cellular pathway causing gut microbiome dysbiosis and the effect of gut microbiome dysbiosis on disease severity. Changes in gut microbiome composition compromise gut barrier function and increase gut permeability, leading to bone loss. Wu et al. reviewed the role of NF-kB inflammatory signaling in osteoporosis. The hyperactivity of inflammatory pathways accelerates osteoporosis and induces osteoclast differentiation. Short-chain fatty acids produced by Bifidobacterium have a beneficial effect on bone mass.

Thank you to all contributing authors and reviewers for their contributions to this topic. We hope everyone enjoys reading this particular Research Topic of Frontiers in Endocrinology and that it inspires and increases understanding of this exciting and growing field of osteo-microbiology.

## Author contributions

AMT: Conceptualization, Formal analysis, Supervision, Writing – original draft, Writing – review & editing. SU: Formal analysis, Investigation, Writing – review & editing. OC: Formal analysis, Investigation, Writing – review & editing.
